# Cost-effectiveness analysis of introducing malaria diagnostic testing in drug shops: A cluster-randomised trial in Uganda

**DOI:** 10.1371/journal.pone.0189758

**Published:** 2017-12-15

**Authors:** Kristian Schultz Hansen, Siân E. Clarke, Sham Lal, Pascal Magnussen, Anthony K. Mbonye

**Affiliations:** 1 Department of Global Health and Development, London School of Hygiene and Tropical Medicine, London, United Kingdom; 2 Department of Public Health, Section for Health Services Research, University of Copenhagen, Copenhagen, Denmark; 3 Department of Disease Control, London School of Hygiene and Tropical Medicine, London, United Kingdom; 4 Centre for Medical Parasitology, University of Copenhagen, Copenhagen, Denmark; 5 Directorate of Clinical and Community Services, Ministry of Health, Kampala, Uganda; 6 School of Public Health, College of Health Sciences, Makerere University, Kampala, Uganda; Johns Hopkins University Bloomberg School of Public Health, UNITED STATES

## Abstract

**Background:**

Private sector drug shops are an important source of malaria treatment in Africa, yet diagnosis without parasitological testing is common among these providers. Accurate rapid diagnostic tests for malaria (mRDTs) require limited training and present an opportunity to increase access to correct diagnosis. The present study was a cost-effectiveness analysis of the introduction of mRDTs in Ugandan drug shops.

**Methods:**

Drug shop vendors were trained to perform and sell subsidised mRDTs and artemisinin-based combination therapies (ACTs) in the intervention arm while vendors offered ACTs following presumptive diagnosis of malaria in the control arm. The effect on the proportion of customers with fever ‘appropriately treated of malaria with ACT’ was captured during a randomised trial in drug shops in Mukono District, Uganda. Health sector costs included: training of drug shop vendors, community sensitisation, supervision and provision of mRDTs and ACTs to drug shops. Household costs of treatment-seeking were captured in a representative sample of drug shop customers.

**Findings:**

The introduction of mRDTs in drug shops was associated with a large improvement of diagnosis and treatment of malaria, resulting in low incremental costs for the health sector at US$0.55 per patient appropriately treated of malaria. High expenditure on non-ACT drugs by households contributed to higher incremental societal costs of US$3.83. Sensitivity analysis showed that mRDTs would become less cost-effective compared to presumptive diagnosis with increasing malaria prevalence and lower adherence to negative mRDT results.

**Conclusion:**

mRDTs in drug shops improved the targeting of ACTs to malaria patients and are likely to be considered cost-effective compared to presumptive diagnosis, although the increased costs borne by households when the test result is negative are a concern.

## Introduction

The private health sector in Africa is an important supplier of treatment for malaria distributing more than half of all antimalarial drugs in many malaria-endemic countries [1,2]. Easy access to antimalarial drugs must be weighed against the risk of substandard practices in a sector often characterised by weak regulation and enforcement in low income countries. Artemisinin-based combination therapies (ACTs) recommended as the first-line treatment for uncomplicated malaria [3] are the most effective but also the most expensive antimalarial on the world market, and sales of cheaper antimalarial monotherapies continue to be common in many countries [4–7]. The effectiveness of the treatment may be further diminished if less than full courses are sold or inadequate instructions are given to patients on how to take the drugs [8–11]. Furthermore, despite the WHO recommendation in 2010 for parasitological confirmation prior to malaria treatment [3], the vast majority of private suppliers do not as yet routinely offer malaria diagnostic testing before selling drugs. A presumptive diagnosis based on clinical signs and symptoms alone will typically result in over-diagnosis of malaria, with recent research showing that more than half of customers purchasing antimalarial drugs from drug shops did not suffer from malaria [1,12,13].

Market interventions to improve malaria treatment in the private sector include the idea of a global subsidy on quality assured ACTs at the factory level introduced as part of the Affordable Medicines Facility—malaria (AMFm) with the expectation that with low price and increased affordability, ACTs would become more widely available in both the public and private sectors, and simultaneously drive out low-priced and less effective antimalarial monotherapies [14]. Pilot evaluations in several countries found that the AMFm subsidy approach achieved considerable success in fulfilling these expectations [15–17]. Subsidising parasitological testing was not part of this initiative despite the possibility that without a test, low priced ACTs might be prescribed to many fever patients not suffering from malaria. Rapid diagnostic tests for malaria (mRDTs) are accurate, easy to use with a quick result, and require limited training and may therefore increase access to parasitological diagnosis in both public and private sectors [1,2,18,19]. There is currently limited experience with subsidising mRDTs in Africa although one study found that introducing mRDTs in Ugandan drug shops increased uptake of testing by customers [20].

The present study was a cost-effectiveness analysis of an intervention to improve the targeting accuracy of sales of subsidised ACTs in Ugandan drug shops by providing drug shop vendor training in mRDT-based diagnosis and access to subsidised mRDTs, compared to drug shops offering only clinical diagnosis and subsidised ACT treatment. At the commencement of the current research, ACT treatment was not subsidised for the private health sector in Uganda. Despite this, subsidised ACTs were introduced in this research both in the intervention and comparator arm as Uganda was one of the AMFm pilot countries [17] and Ministry of Health considered making ACT subsidisation permanent.

## Methods

### Study area and population

A cluster-randomised trial was conducted in registered drug shops in Mukono District, Central Uganda; an area with perennial malaria transmission and with a fever prevalence of 42% among children below 5 years in 2011 [21]. The majority of the population live in rural areas and are predominantly subsistence farmers. Registered drug shops are licensed to sell non-prescription drugs, including antimalarial drugs but not antibiotics or injections. At the beginning of the present study, parasitological diagnosis was not commonly available in drug shops, although mRDTs or microscopy were available at government health facilities [5].

### Intervention

The intervention consisted of training drug shop vendors to perform and sell subsidised mRDTs and advise on the purchase of ACTs in accordance with national treatment guidelines, and at low subsidised prices. Participating drug shops received these commodities for free, to be sold at agreed recommended retail prices in 2011 of US$0.20 for an mRDT and US$0.40–1.19 for a course of ACT depending on age, as informed by a prior willingness-to-pay study [22]. Drug shops in the control arm also received ACTs for free and offered these to customers at the same subsidised prices, but based on clinical diagnosis only. All drug shops in both arms treated customers with signs of severe malaria with rectal artesunate without charge and subsequently recommended referral. Details of the training intervention and supporting activities are available online (www.actconsortium.org/RDTdrugshops) and in prior publications [23,24]. In brief, all drug shop vendors attended a 3-day participatory training workshop on malaria case management, including training on the signs and symptoms of malaria and administration of ACT or rectal artesunate (depending on severity), with drug shops in the intervention arm receiving an additional day of training on how to perform and interpret mRDT diagnostic tests. This was followed by close support supervision with site visits for the first three months of implementation after which supervision was scaled down considerably. Community sensitisation was also carried out to inform the population about the upcoming study, the benefits of mRDT testing, and the availability of mRDTs from local health facilities and trained drug shop vendors. In summary, pricing of commodities, training and supporting activities were identical between the two study arms except that drug shops in the intervention arm were allowed to sell subsidised mRDTs based on one additional day of training.

### Measurement of effect

The primary aim of the trial was to evaluate the effect of mRDT diagnostic testing on the accuracy of targeting of ACTs. Antimalarial treatments sold by drug shop vendors were thus validated by expert microscopy on a blood slide collected by the vendor from the customer at the time of consultation and read later by the research team (reference diagnosis). The measure of effect was ‘appropriate treatment of malaria with ACT or rectal artesunate (depending on severity of symptoms)’—a composite indicator defined as: a patient with a positive reference diagnosis of malaria purchasing a course of ACT (or receiving rectal artesunate for free) or a patient with a negative reference diagnosis not sold an ACT (or receiving rectal artesunate).

The proportion of patients appropriately treated of malaria with ACT or rectal artesunate in each study arm was obtained from a cluster-randomised trial in Mukono District [24]. A cluster was defined as a natural grouping of registered drug shops in close geographical proximity. Twenty clusters were randomised either to diagnosis by mRDT or diagnosis by clinical signs and symptoms, with 10 clusters in each arm. Informed consent to participate in the trial was obtained from drug shop vendors prior to training. For every customer seeking treatment for fever, drug shop vendors recorded the diagnosis, mRDT result (intervention arm only), and any ACT treatment sold in a register designed for this purpose. Vendors were not asked to routinely record other drugs sold. Appropriate treatment of patients for malaria with ACT or rectal artesunate was measured over a 12-month period from January-December 2011 where 7522 customers were recruited in the intervention arm and 5797 in the control arm [24]. (There were very few rectal artesunate treatments (<1%). For the remainder of the paper, ACT or rectal artesunate depending on severity of symptoms are therefore simply referred to as ‘ACT’).

### Measurement of costs

Economic costs of resources were measured from both the public health sector and societal perspectives and presented separately. All cost figures were adjusted to the 2011 price level using an annual inflation rate of 8.8% corresponding to the average annual increase in the GDP deflator in Uganda from 2004 to 2013 and presented in US dollars (UGX2523 = US$1) [25].

The total costs of resources used for training of drug shop vendors, supervision, and community sensitisation were considered a health sector cost. Personnel costs of these three activities were captured through interviews with participating staff who were asked to estimate days utilised and about their gross monthly salary. All other costs were obtained from project financial accounts that had been maintained throughout the study period. Training of drug shop vendors, an initial period of close support supervision and community sensitisation were expected to have a longer useful lifespan beyond the evaluation period of the 2011 calendar year and were treated as capital goods with annual equivalents calculated assuming a lifespan of 5 years and a real discount rate of 3% [26]. As all mRDTs and courses of ACT and rectal artesunate were supplied free to drug shops, the cost of acquiring these was also considered a health sector cost. The mRDT utilised in this study, First Response, was available in Uganda in 2011 at US$0.87 per test, including shipping and quality control. Adding an assumed test wastage rate of 5%, as well as the costs of estimated consumption of sterile gloves, cotton wool, and spirit, this resulted in a unit cost of US$1.00 per mRDT performed. The median prices per course of ACT treatment with artemether-lumefantrine and rectal artesunate suppository were obtained from an international drug price list [27]. A recommended ten percent was added for shipping [27] and another 12.5% added to cover storage and quality control in-country (personal communication, Central Medical Stores). Assuming 10% wastage, the estimated cost per course of ACT in 2011 ranged from US$0.77 for a child below 3 years of age to US$2.25 for an adult treatment dose and US$0.74 for a rectal artesunate suppository.

Household costs were captured for a two-week period following the visit to a drug shop in a random sample of 506 drug shop customers and interviewed at home 4 days after their visit to the drug shop followed by a second home visit between days 10–14. Interviewers visited drug shops participating in the cluster-randomised trial [24] according to a randomised schedule to identify recent customers from the registers kept by drug shop vendors. Interviewers inquired from the patient, or main caregiver in case of a child, all relevant out-of-pocket expenditure on transport, drugs, consultation fees, diagnostic tests, special foods purchased to aid recovery, incurred at the time of the initial visit to a drug shop, and as well as during any subsequent treatment seeking visits to any health provider. Respondents were also asked about the number of days they were unable to perform their normal activities due to illness. Lost time was valued at US$1.21 per day corresponding to the GDP per capita per day in 2011 [25].

The sample size for household cost interviews was calculated to test a hypothesis that out-of-pocket expenditure would be lower in the intervention arm. To detect a decrease of at least 30% in mean out-of-pocket expenditure in the intervention arm from a mean cost of UGX3500 in the control arm as informed by previous, unpublished research in Mukono District, and assuming k = 0.25, power of 80%, a significance level of 5% in a trial with 10 clusters per arm, required 250 interviews per arm [28].

### Incremental cost-effectiveness analysis

**Fig 1:**
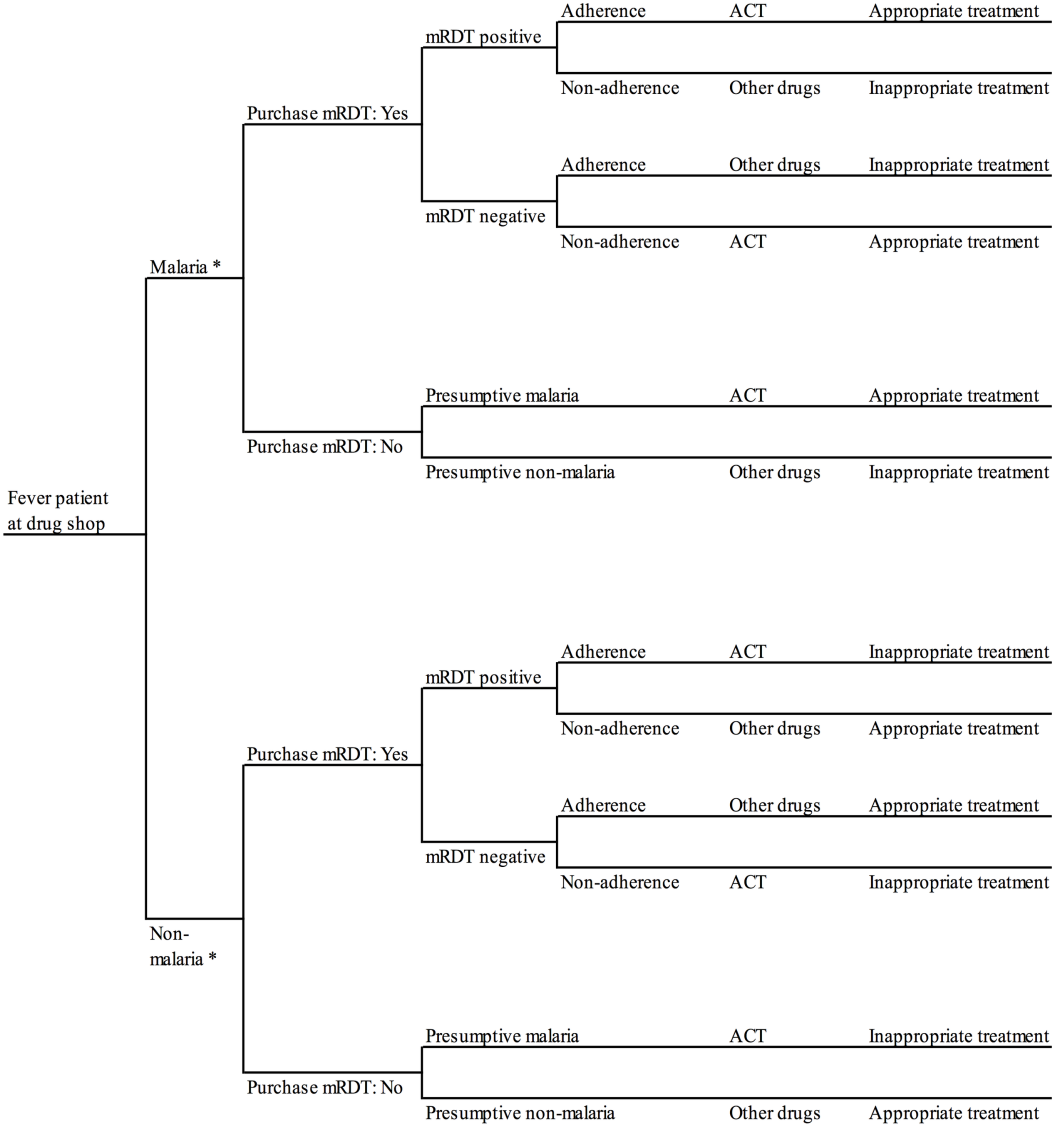
Decision model for customers visiting drug shops offering mRDT diagnosis, Mukono District, Uganda. * According to expert microscopy on a blood slide collected the by drug shop vendor from the customer at the time of consultation and read later by the research team (reference diagnosis).

**Fig 2:**
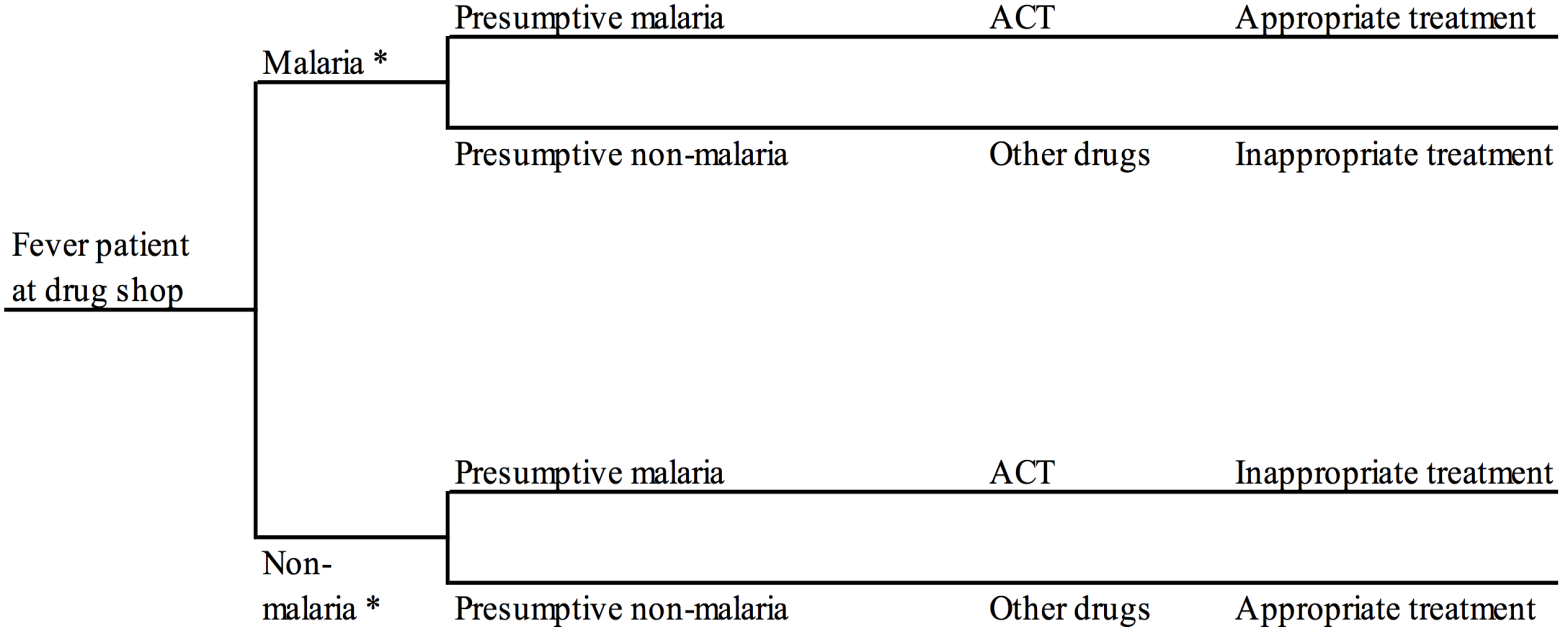
Decision tree for customers visiting drug shops offering presumptive diagnosis, Mukono District, Uganda. * According to expert microscopy on a blood slide collected the by drug shop vendor from the customer at the time of consultation and read later by the research team (reference diagnosis).

A decision analysis approach [29,30] was used to link data on cost and effects from the different primary data collection activities conducted in Mukono District described above. According to the decision tree utilised for the mRDT arm (Fig 1), a customer visiting a drug shop offering parasitological diagnosis must first decide whether to purchase an mRDT or not followed by a decision by customer and vendor to adhere to the test result and choose what drugs to purchase. A simpler decision tree was used for the presumptive arm (Fig 2). All event probabilities required for the decision tree in each arm were obtained from the trial [24] except the probability of additional treatment-seeking following the drug shop visit which was captured from the subsample of household cost interviews. Total health sector and societal costs by study arm were calculated by populating the decision trees with unit cost estimates per drug shop visit. The annualised 2011 costs of training of drug shop vendors, supervision, and community sensitisation by study arm were divided by the number of customer visits enrolled in the trial during the 2011 calendar year arriving at the average unit cost per visit for these three activities. Estimated costs per mRDTs and ACTs supplied to drug shops and borne by the health sector included the prices of commodities themselves, transport, quality control and other required supplies (see the previous section). Patients’ out-of-pocket expenditure on mRDTs and ACT treatments purchased during drug shop visits were assumed to equal the recommended retail price of these commodities in the trial period. Responses from the 506 household cost interviews suggested that this was a fair assumption. Household cost per visit of other drugs, fees, food, transport, and time lost during the initial and subsequent visits were obtained from the household interviews. All event probabilities and cost input used to populate the decision trees for the cost-effectiveness analysis are listed in Table 1.

**Table 1:** Parameters utilised in decision model and distributions for probabilistic sensitivity analyses (PSA), incremental cost-effectiveness analysis of replacing presumptive diagnosis by rapid diagnostic tests in drug shops in Mukono District, Uganda, 2011 (US$1 = UGX2523).

Model parameter	---------- Value ----------		Source	Distribution in PSA
	mRDT arm	Presumptive arm		
Malaria positivity rate among customers suspected of malaria (%) ^#^	43.49	31.76	[24]	Point estimate
Share of customers suspected of malaria purchasing mRDT (%)	99.95	NA	[24]	Beta
Sensitivity of diagnosis (%)	91.75	99.89	[24]	Beta
Specificity of diagnosis (%)	62.92	0.20	[24]	Beta
Adherence to positive mRDT result (%)	99.10	NA	[24]	Beta
Adherence to negative mRDT result (%)	98.60	NA	[24]	Beta
Community sensitisation, cost per visit (US$)	0.12	0.15	*	Point estimate
Training of drug shop vendors, cost per visit (US$)	0.57	0.61	*	Point estimate
Supervision, cost per visit (US$)	0.47	0.48	*	Point estimate
Cost per mRDT, paid by the health sector (US$)	1.00	NA	§	Point estimate
Cost per ACT course (< 3 years) paid by the health sector (US$)	0.77	0.77	§	Point estimate
Cost per ACT course (3–7 years) paid by the health sector (US$)	1.63	1.63	§	Point estimate
Cost per ACT course (8–14 years) paid by the health sector (US$)	1.98	1.98	§	Point estimate
Cost per ACT course (> 14 years) paid by the health sector (US$)	2.25	2.25	§	Point estimate
Cost per rectal artesunate paid by the health sector (US$)	0.74	0.74	§	Point estimate
Price per mRDT in drug shops (US$)	0.20	NA	&	Point estimate
Price per ACT course (< 3 years) in drug shops (US$)	0.40	0.40	&	Point estimate
Price per ACT course (3–7 years) in drug shops (US$)	0.59	0.59	&	Point estimate
Price per ACT course (8–14 years) in drug shops (US$)	0.59	0.59	&	Point estimate
Price per ACT course (> 14 years) in drug shops (US$)	1.19	1.19	&	Point estimate
Price per rectal artesunate in drug shops (US$)	0.00	0.00	&	Point estimate
Out-of-pocket expenditure for non-ACT drugs per visit (US$)	2.08	1.10	€	Gamma
Out-of-pocket expenditure for fees, transport, etc per visit (US$)	0.58	0.56	€	Gamma
Probability of additional treatment-seeking (%)	9.82	10.22	€	Beta
Out-of-pocket expenditure per additional visit (US$)	2.58	1.84	€	Gamma
Time utilised for travelling and waiting per fever episode (days)	0.10	0.10	€	Gamma
Time unable to perform normal activities per fever episode (days)	2.11	1.80	€	Gamma
Value of lost time per day (US$)	1.21	1.21	[25]	Point estimate

^#^ According to expert microscopy on a blood slide collected by the drug shop vendor at the time of consultation and blind reading later by the research team (reference diagnosis).

* According to study accounting system (see text).

^§^ Including price of commodity, transport, disposables and waste (see text).

^&^ Recommended retail price agreed with drug shop vendors participating in the trial.

^€^ Sample of household cost interviews (see text).

Total health sector and societal costs and number of patients appropriately treated of malaria were calculated for a standard population of 1000 drug shop customers in each study arm using the populated decision trees. The incremental cost-effectiveness ratio (ICER) was estimated by dividing the difference in the total costs in the two arms by the difference in the number of patients appropriately treated of malaria in the two arms. The ICER represented the cost per additional drug shop customer appropriately treated of malaria if subsidised mRDTs were introduced into drug shops that previously diagnosed malaria presumptively.

### Sensitivity analyses

Univariate sensitivity analyses were performed to assess the robustness of the estimate of the ICER of introducing mRDTs into drug shops to relevant parameters including malaria prevalence among drug shop customers, accuracy of the mRDT and adherence to test results. As malaria prevalence and number of customers were both found to be higher in the mRDT arm as compared to the presumptive arm in 2011 [24], a scenario analysis assuming the same level (equal to the average) for these parameters in both arms was performed to assess the impact of this imbalance.

Probabilistic sensitivity analysis (PSA) was developed to estimate the combined influence of sampling uncertainty in relevant parameters [29] using beta distributions for the event probabilities in the decision trees and gamma distributions for all household cost parameters (Table 1). Random and simultaneous selection of values from these parameter distributions, followed by calculation of ICERs [31], was performed 10000 times in Excel (Microsoft, Seattle). The resulting estimated uncertainty of the ICERs was summarised by plotting joint incremental costs and incremental effects from replacing presumptive diagnosis by mRDTs in the cost-effectiveness plane and using these scatter plots to derive cost-effectiveness acceptability curves (CEACs). These curves were derived by calculating the proportion of pairs of incremental cost and effects and consequent ICERs where the introduction of mRDTs would be considered cost-effective given different levels of willingness-to-pay (WTP) of the health policy maker per appropriately treated patient for malaria [32–34].

### Ethical statement

Ethical approval for the research was granted by review boards at the Uganda National Council of Science and Technology and London School of Hygiene and Tropical Medicine. Written informed consent was obtained from drug shop vendors to participate in the trial and from the patient (or caregiver) prior to household interviews. Verbal consent was sought from patients at the time of drug shop consultation for an mRDT and/or research blood slide.

## Results

The main results of this research are presented in Table 2 for a standard population of 1000 customers in each of the two study arms. The introduction of mRDTs in the intervention arm resulted in a significant increase in the number of fever patients appropriately treated of malaria compared to the presumptive arm—751 versus 319 customers or an increase of 433 per 1000 febrile customers seen (95% CI 424 to 442). The ICER from a health sector perspective was US$0.55 (95% CI US$0.51 to US$0.60) meaning that introducing subsidised mRDT diagnosis in drug shops currently offering presumptive treatment with subsidised ACT would cost the health sector US$0.55 per additional patient appropriately treated of malaria. Applying a broader societal perspective, which includes health sector and household costs, the incremental cost of introducing mRDTs was US$3.83 per additional patient appropriately treated of malaria (95% CI -US$23.87 to US$30.81).

**Table 2:** Costs and effects in a standard population of 1000 individuals suspected of malaria by study arm and incremental cost-effectiveness ratio (ICER) of replacing presumptive diagnosis by rapid diagnostic tests in drug shops in Mukono District, Uganda, 2011 (US$1 = UGX2523).

	--- mRDT arm ---		-- Presumptive arm --	
	***N***	***%***	***N***	***%***
*Individuals suspected of malaria*	*1000*	*100*	*1000*	*100*
True malaria ^#^	435	43	318	32
Purchased ACT	609	61	998	100
Appropriately treated *	751	75	319	32
	***US$***	***%***	***US$***	***%***
*Health sector cost per 1000 individuals*	*3129*	*33*	*2890*	*37*
Community sensitisation	116	1	148	2
Training of vendors	572	6	608	8
Supervision of vendors	470	5	482	6
mRDTs	999	11	0	0
ACTs	973	10	1651	21
*Household cost per 1000 individuals*	*6287*	*67*	*4868*	*63*
mRDTs (first visit)	198	2	0	0
ACTs (first visit)	435	5	734	9
Other drugs (first visit)	2164	23	1101	14
Fees, travel, food (first visit)	581	6	562	7
Drugs, fees, travel, food (subsequent visits)	254	3	188	2
Opportunity cost of time lost	2655	28	2283	29
*Total societal cost per 1000 individuals*	*9415*	*100*	*7757*	*100*
**Incremental analysis (Replace presumptive diagnosis by mRDT in 1000 individuals suspected of malaria)**				
Incremental number of appropriately treated [95% CI]		433	[424; 442]	
Incremental health sector cost, US$ [95% CI]		239	[224; 254]	
Incremental societal cost, US$ [95% CI]		1658	[-10350; 13254]	
ICER health sector perspective, US$ [95% CI]		0.55	[0.51; 0.60]	
ICER societal perspective, US$ [95% CI]		3.83	[-23.87; 30.81]	

^#^ According to expert microscopy on a blood slide collected by the drug shop vendor at the time of consultation and blind reading later by the research team (reference diagnosis).

* Individual with a positive reference diagnosis of malaria purchasing a course of ACT or an individual with a negative reference diagnosis not purchasing an ACT.

The absolute health sector costs were similar in the two arms, at US$3.13 per drug shop customer with suspected malaria seen in the mRDT arm and US$2.89 in the presumptive arm (Table 2)–a difference of US$0.24 per customer. These were the average costs per customer suspected of malaria for training, supporting interventions, and commodities (ACTs and diagnostics) by arm to be financed by the Ministry of Health. Health sector costs constituted 33% and 37% of total societal costs in the mRDT and presumptive arms respectively. The most important health sector cost component was ACTs consumed forming 10% and 21% of total societal cost in the two arms and mRDTs utilised which constituted 11% in the mRDT arm. The sum of absolute cost of mRDTs and ACTs was higher in the mRDT arm than in the presumptive arm meaning that the extra cost of supplying mRDTs was only partially offset by a reduction in the consumption of ACTs.

Societal cost was considerably higher at US$9.42 and US$7.76 per customer with suspected malaria in the mRDT and presumptive arms respectively (Table 2)–a difference of US$1.66 per customer. Household costs formed 67% and 63% of total societal costs in the mRDT and presumptive arms respectively. Out-of-pocket expenditure on drugs other than ACTs (such as antipyretics, various injections, antibiotics, and cough syrups) formed an important part of household cost, and non-ACT drug expenditure was twice as high among customers visiting mRDT drug shops as compared to customers visiting drug shops offering presumptive diagnosis. Only ten percent of households in the sample reported seeking additional care after the first visit to a drug shop and similar in both arms (9.8% versus 10.2%) indicating that diagnostic testing in drug shops did not have a marked effect on subsequent treatment seeking behaviour (Table 1). Household costs related to additional health care seeking formed only 2% of total societal cost in both arms. Opportunity costs of lost time were also similar constituting close to one third of total societal cost in both study arms. Days lost due to illness or caring for an ill family member were the most important component of opportunity costs of lost time.

The univariate sensitivity analyses suggested that the ICER of introducing mRDTs from both the health sector and societal perspectives was robust except when selected parameters were markedly different from their central values (Table 3). If the malaria prevalence among drug shop customers had been 60% instead of 43% and 32% as found in the mRDT and presumptive arms, then the ICER from a health sector perspective increased from US$0.55 to US$1.92. At malaria prevalence levels of below 16% the ICER was negative meaning that the mRDT had lower health sector cost and higher effect than presumptive diagnosis. A lower mRDT price, increased mRDT specificity, and higher popularity of drug shops offering mRDTs would improve the cost-effectiveness of mRDT introduction while decreased adherence to mRDT results and lower ACT prices decreased cost-effectiveness. All other parameters and assumptions incorporated in the univariate sensitivity analysis did not have strong influence on the ICER levels.

**Table 3:** Sensitivity to selected parameters of the incremental cost-effectiveness ratio (ICER) of replacing presumptive diagnosis by rapid diagnostic tests in drug shops in Mukono District, Uganda, 2011 (US$1 = UGX2523).

Parameter ^&^	--- ICER in US$ ---		Parameter ^&^	--- ICER in US$ ---	
	Health sector	Societal		Health sector	Societal
***Malaria prevalence among customers (43% and 32%)***			***Prob*. *of subs*. *treatment in mRDT arm (9*.*8%)***		
10%	-0.09	2.72	2%	0.55	3.34
20%	0.08	3.21	20%	0.55	4.47
40%	0.61	4.82	35%	0.55	5.40
60%	1.92	8.72	***Prob*. *of subs*. *treatment in presumptive arm (10*.*2%)***		
80%	9.72	32.03	2%	0.55	4.20
***Sensitivity of mRDT (92%)***			20%	0.55	3.39
70%	0.28	4.61	35%	0.55	2.71
85%	0.48	4.04	***ACT price***		
100%	0.63	3.61	30% decrease	1.02	4.30
***Specificity of mRDT (63%)***			40% decrease	1.18	4.46
50%	0.98	4.75	50% decrease	1.34	4.61
70%	0.37	3.44	***mRDT price (US$0*.*70)***		
90%	0.00	2.63	30% decrease	-0.08	3.20
100%	-0.14	2.33	40% decrease	-0.29	2.99
***Adherence to negative mRDT (99%)***			50% decrease	-0.50	2.78
40%	2.49	7.61	***Discount rate (3%)***		
60%	1.56	5.81	1%	0.56	3.84
80%	0.96	4.63	7%	0.54	3.82
***Change in number of customers in mRDT arm***			10%	0.53	3.81
40% lower	2.21	5.49	***Com*. *sens*., *training and intense sup*. *(every 5 years)***		
40% higher	-0.16	3.12	Every 3 years	0.49	3.77
80% higher	-0.55	2.73	Every 7 years	0.58	3.86
100% higher	-0.69	2.59	***Opportunity cost per day (US$1*.*2)***		
***Change in number of customers in presumptive arm***			US$0.4	0.55	3.26
40% lower	-1.23	2.05	US$0.8	0.55	3.54
40% higher	1.32	4.60	US$1.6	0.55	4.11
80% higher	1.74	5.02	US$2.0	0.55	4.39
100% higher	1.89	5.17	US$2.5	0.55	4.75

^&^ Actual parameter value observed in the trial [24] is shown in parenthesis.

In the scenario analysis, assuming identical malaria prevalence and number of clients in 2011 with suspected malaria in mRDT and presumptive diagnosis drug shops, the ICER increased to US$1.33 from a health sector perspective and to US$5.42 from a societal perspective (S1 Table). This increase was due to a larger difference in health sector cost between the study arms and a smaller difference in the number of patients appropriately treated of malaria compared to the central estimates utilised in Table 2.

**Fig 3:**
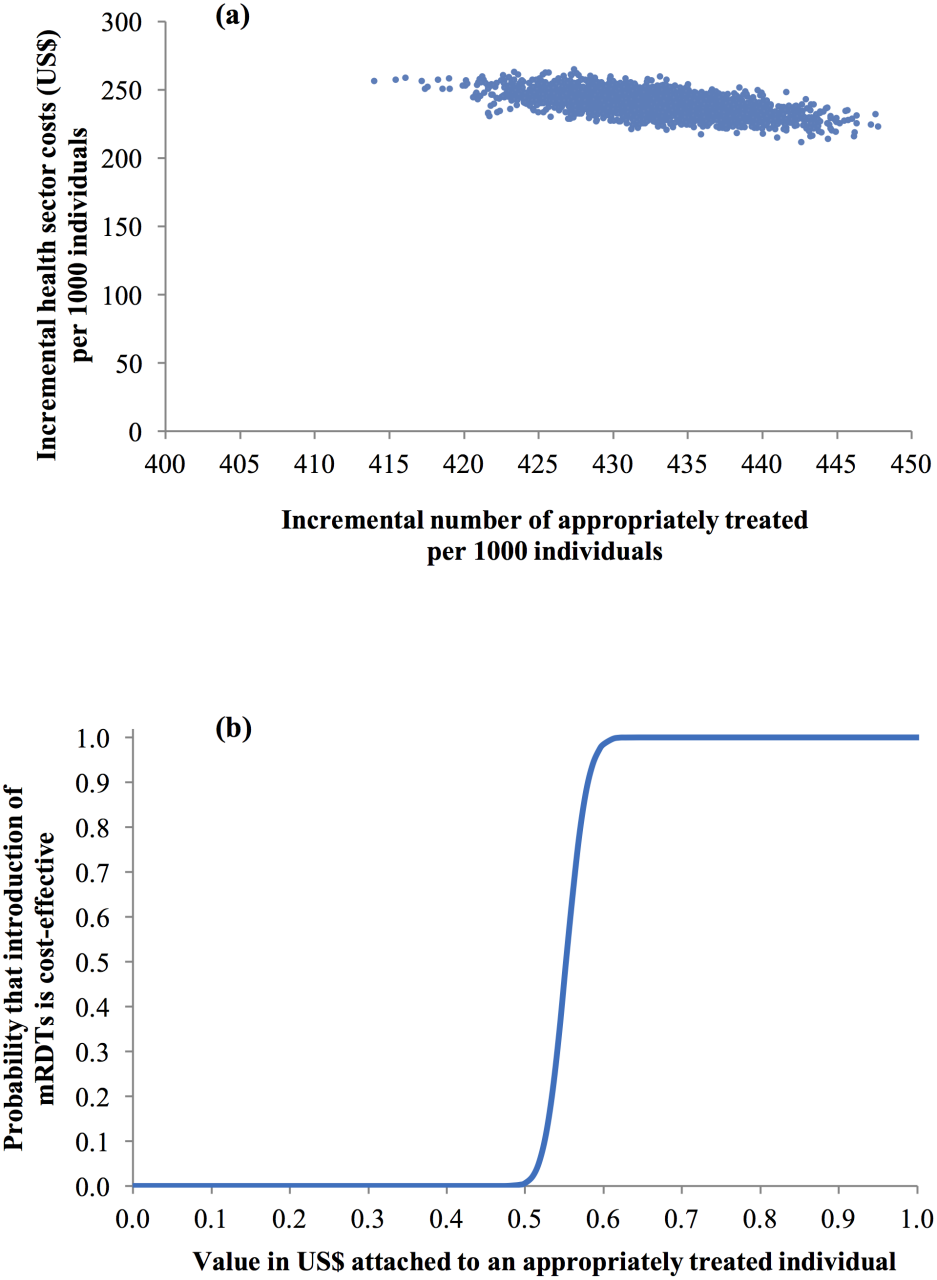
Probabilistic sensitivity analysis (health sector perspective). (a) scatter plot of incremental health sector cost in US$ and incremental number of individuals appropriately treated of malaria resulting from replacing clinical diagnosis of malaria by rapid diagnostic test in drug shops, Mukono District, Uganda, 2011 (US$1 = GHS1.51) and (b) cost-effectiveness acceptability curve.

The probabilistic sensitivity analysis (PSA) from a health sector perspective found that all iterations led to positive incremental health sector costs and a positive incremental number of individuals appropriately treated of malaria in the cost-effectiveness plane (Fig 3a). Shifting from presumptive to mRDT diagnosis therefore resulted in a significant increase in the number of appropriately treated individuals with a mean of 433 (95% CI 424 to 442) out of 1000 drug shop customers and a significant increase in health sector cost with mean US$239 per 1000 (95% CI US$224 to US$254). Using this scatterplot to develop the CEAC by calculating the share of the pairs of incremental costs and effects leading to ICERs below given threshold values (Fig 3b), it was found that if a policy maker was willing to pay (WTP) at least US$0.55 per patient appropriately treated of malaria, the probability of mRDT introduction being a cost-effective intervention was 46%. This probability increased markedly to 79% and 100% if WTP was US$0.57 and US$0.61 or above. There was therefore a high probability that the introduction of mRDTs would be cost-effective even at very low WTP.

**Fig 4:**
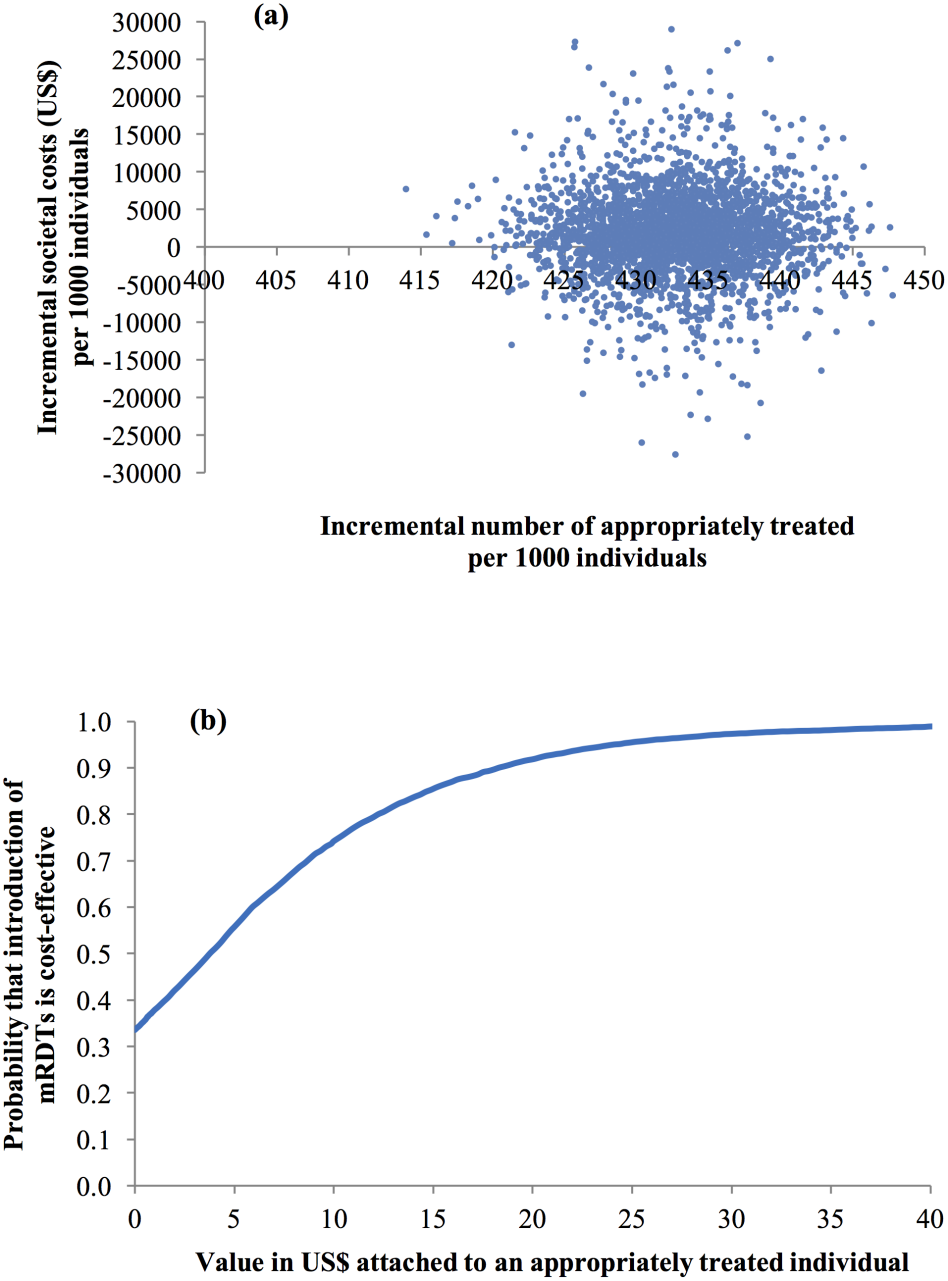
Probabilistic sensitivity analysis (societal perspective). (a) scatter plot of incremental societal cost in US$ and incremental number of individuals appropriately treated of malaria resulting from replacing clinical diagnosis of malaria by rapid diagnostic test in drug shops, Mukono District, Uganda, 2011 (US$1 = GHS1.51) and (b) cost-effectiveness acceptability curve.

The PSA from a societal perspective revealed a higher degree of uncertainty associated with making a decision with respect to the introduction of mRDTs. The scatterplot of joint incremental societal cost and incremental number of individuals appropriately treated of malaria in the cost-effectiveness plane (Fig 4a) exhibited considerable variation in costs being sometimes positive and sometimes negative with a mean of US$1658 per 1000 customers (95% CI –US$10350 to US$13254). The CEAC revealed that much higher WTP was required to deem the introduction of mRDTs as a cost-effective intervention. If the WTP was US$1, the probability of mRDT introduction being cost-effective was 38%; increasing to 56%, 74%, and 92% if the WTP was US$5, US$10, and US$20 respectively (Fig 4b).

## Discussion

The present study compares the cost and effects of introducing subsidised mRDT-based diagnosis versus presumptive diagnosis in private drug shops in Uganda selling subsidised ACTs. This design is appropriate for a situation where subsidised ACTs have already been introduced and where a decision must now be made if it would be cost-effective also to introduce subsidised mRDTs in private drug shops. From a health sector perspective, the introduction of mRDTs in drug shops is likely to be considered a highly cost-effective intervention since the cost to the health sector per extra appropriately treated client of malaria was low at only US$0.55. For comparison, this was substantially lower than the range of health sector cost of US$5.0 to US$10.5 per appropriately treated patient of introducing mRDTs in public health centres recently found in different African countries [35–37]. The main explanations for the very low ICER of introducing subsidised mRDTs in drug shops were the substantial improvement in the appropriate targeting of subsidised ACT treatment to customers infected with malaria parasites (43% points) and the fact that the comparator, presumptive treatment with subsidised ACTs, involved similar levels of training, supervision and community sensitisation and therefore costs as the mRDT intervention. Although mRDT use in drug shops did not result in a net cost saving for the government, they helped to ensure that subsidised ACTs were more accurately targeted at minimal additional cost (US$0.24 per customer suspected of malaria). The sensitivity analyses performed did not give reason to change this conclusion except at high malaria prevalence among customers, low adherence to negative mRDTs, and lower ACT price where the introduction of mRDTs would be increasingly less cost-effective relative to presumptive diagnosis.

From a wider societal perspective, it was found that the total societal cost of introducing mRDTs was considerably higher and estimated at US$3.83 per additional appropriately treated patient due to the high proportion of costs borne by households. The variation in household costs as captured during household interviews was large and right-skewed with the standard deviation being at a similar level or higher than the mean cost of all household cost categories. This is however not an uncommon pattern in cost data [29,38]. Consequently, the decision to introduce mRDTs is also more uncertain from a societal perspective as the probability of mRDTs being a cost-effective intervention only exceeds 75% if a high value (more than US$10) is attached to each additional customer appropriately treated of malaria. From a narrow health sector perspective, mRDTs would be deemed cost-effective with a probability of 75% at much lower WTP of US$0.57.

Our results underline the large share of societal cost that was borne by households seeking treatment in the private sector, even when subsidised ACTs were available, and important explanations were extensive purchasing of non-ACT drugs as well as the opportunity cost of lost time. Non-ACT drug expenditure was almost twice as high among customers who visited drug shops with mRDT diagnosis compared to customers attending drug shops with clinical diagnosis and total out-of-pocket expenditure on drugs was thus 41% higher among customers attending drug shops that offered mRDT-based diagnosis. Another important explanation of high household cost was opportunity cost of lost time where patients were unable to do their normal activities. Valuing lost time per day by the GDP per capita might be an overestimate for a population consisting predominantly of subsistence farmers but assuming a lower value of lost time did not have a strong influence on the ICER level.

A key parameter to judging the introduction of mRDTs a cost-effective intervention or not was adherence to negative mRDT results. The trial found a very high adherence to negative mRDT results by customers and vendors [24]. Diagnostic testing appeared to enhance the reputation of drug shops and may have increased custom [39], potentially increasing the desire of vendors to comply with the intervention. Another contributing factor to this high adherence could be that negative mRDTs did not just represent a loss in drug shop vendor income due to lower antimalarial drug sales but also an opportunity to sell a range of other drugs. In this setting, there appeared not to be a strong financial incentive to disregard negative mRDT results on the part of the vendors.

The availability of mRDTs in drug shops led to a significant decrease in ACT treatment sold to patients with no malaria parasites in their blood from almost 100% in the presumptive arm to 37% in the mRDT arm. National roll-out of mRDTs in the private retail sector may therefore contribute to delayed emergence and spread of artemisinin resistance. If such longer term positive effects had been included in the analysis, this would have improved the cost-effectiveness of mRDTs relative to presumptive diagnosis. In addition, mRDTs ensured that many more customers with non-malarial fever were correctly informed that they did not have malaria (close to 0% in the presumptive arm and 63% in the mRDT arm). Such knowledge may improve the chances that these non-malarial febrile illnesses are correctly treated as patients may start searching for an alternative, correct diagnosis and treatment. Although this aspect was not formally investigated as part of this research project, improved detection and treatment of non-malarial febrile illnesses would make mRDT introduction more cost-effective.

This research also pointed to some risks related to the introduction of mRDTs in drug shops. Among the malaria parasite-free patients as judged by expert microscopy, 37% of these were deemed positive by mRDT and almost all of these patients purchased an ACT. Some of these patients may not be treated for other causes of fever as both DSV and patient may regard ACT as sufficient treatment for the fever. Similarly, as Mukono District is an area with perennial malaria transmission, some mRDT positive patients may be asymptomatic carriers of malaria with other co-infections and again may not be treated immediately for the other causes of fever, thus increasing the risk of adverse health for patients.

The shift in pattern of medicines purchased in favour of non-ACT drugs among customers visiting drug shops offering mRDT may be appropriate given the higher percentage of patients correctly diagnosed as not suffering from malaria. However, there may also be an increased risk that following a negative mRDT, DSVs diagnose and treat conditions they are not properly trained for again potentially elevating the risk to patients.

There were no reported deaths among the sample of 506 customers interviewed in their homes and only 3% of the customers visiting an mRDT drug shop answered during the interview that they were still ill on day 14. Nevertheless, additional research would be desirable to investigate the extent of the risks identified above as an input into the decision whether and how mRDTs should be introduced into the private retail sector.

## Limitations

Household costs of non-ACT drugs were captured through the interviews with customers. There may be several sources of error associated with self-reported household costs during interviews including recall bias with respect to types and prices of drugs purchased. In addition, it was only possible to perform a limited number of home interviews. As a result, the household costs for non-ACT drugs may have been determined with higher uncertainty than the household costs of ACT.

The trial was performed in registered drug shops, and there were other sources of malaria treatment within the study area including public facilities and private clinics, as well as unregistered drug shops. Thus although we cannot rule out the possibility of differential treatment-seeking by customers according to the method of diagnosis available in different drug shops, which may account for the differing prevalence of infection between the two study arms, we consider this difference to reflect a realistic situation of provider choice. Thus, although we also present results from a scenario analysis assuming equal prevalence and attendance in drug shops by diagnostic method for comparison, we would contend that the central estimates are a more realistic and thus generalisable assessment of the cost-effectiveness of introducing mRDTs into private drug shops.

Ministry of Health staff were closely involved in the design of the intervention to ensure that the training and supervision approach used in the trial would be feasible to replicate at scale. Nevertheless, the effects of the intervention were estimated under relatively controlled conditions, and comparable effects and cost-effectiveness could prove harder to achieve under routine operational conditions.

The focus of this research was the ‘appropriate treatment of malaria with ACT’. The appropriateness of treatment of customers not having malaria could not be investigated as this would have required an independent clinical assessment of the patient, which was beyond the scope of this trial. It was therefore not possible to judge if the household expenditure on non-ACT drugs were well spent in terms of having a positive medical benefit. The analysis examined only the immediate costs and effects of the intervention, and did not consider potential longer-time economic benefits that could arise, such as improved detection and treatment of other non-malaria febrile illnesses, and delayed emergence and spread of drug resistance due to reduced drug pressure.

## Conclusions

The present research suggests that the introduction of subsidised mRDTs in private drug shops in Uganda is desirable from a pure cost-effectiveness perspective compared to a situation with presumptive diagnosis. It was found that the availability of this parasitological test in drug shops significantly increased the proportion of patients appropriately treated of malaria (from 32% to 75%) at a low incremental cost of US$0.55 per appropriately treated patient from a health sector perspective and US$3.83 from a societal perspective. Furthermore, the additional cost per suspected malaria patient being offered subsidised mRDT and ACT was only US$0.24 to be covered by the health sector and US$1.66 for the society as a whole compared to customers purchasing presumptive diagnosis and subsidised ACT. The increased costs borne by households when the test result is negative are a potential concern, and additional research would be helpful to investigate if the medicines purchased by patients diagnosed not to suffer from malaria are appropriate, have sufficient clinical benefit and represent good value for money.

## Supporting information

S1 Table Scenario analysis assuming identical malaria positivity rate and number of customers by study arm: Costs and effects in a standard population of 1000 individuals suspected of malaria by study arm and incremental cost-effectiveness ratio (ICER) of replacing presumptive diagnosis by rapid diagnostic tests in drug shops in Mukono District, Uganda, 2011 (US$1 = UGX2523). (DOCX)

## References

[1] WHO. World Malaria Report 2012. Switzerland: World Health Organization; 2012.

[2] O’Connell KA, Gatakaa H, Poyer S, Njogu J, Evance I, Munroe E, et al Got ACTs? Availability, price, market share and provider knowledge of anti-malarial medicines in public and private sector outlets in six malaria-endemic countries. Malar J. 2011; 10: 326 doi: 10.1186/1475-2875-10-326 22039838 10.1186/1475-2875-10-326 PMC3227612

[3] WHO. Guidelines for the treatment of malaria, 2nd edition Switzerland: World Health Organization; 2010.

[4] Littrell M, Gatakaa H, Evance I, Poyer S, Njogu J, Solomon T, et al Monitoring fever treatment behaviour and equitable access to effective medicines in the context of initiatives to improve ACT access: baseline results and implications for programming in six African countries. Malar J. 2011; 10: 327 doi: 10.1186/1475-2875-10-327 22039892 10.1186/1475-2875-10-327 PMC3223147

[5] Mbonye AK, Lal S, Cundill B, Hansen KS, Clarke S, Magnussen P. Treatment of fevers prior to introducing rapid diagnostic tests for malaria in registered drug shops in Uganda. Malar J. 2013; 12: 131 doi: 10.1186/1475-2875-12-131 23587179 10.1186/1475-2875-12-131 PMC3637132

[6] Palafox B, Patouillard E, Tougher S, Goodman C, Hanson K, Kleinschmidt I, et al Understanding private sector antimalarial distribution chains: a cross-sectional mixed methods study in six malaria-endemic countries. PLoS One. 2014; 9: e93763 doi: 10.1371/journal.pone.0093763 24699934 10.1371/journal.pone.0093763 PMC3974780

[7] Mangham-Jefferies L, Hanson K, Mbacham W, Onwujekwe O, Wiseman V. Mind the gap: knowledge and practice of providers treating uncomplicated malaria at public and mission health facilities, pharmacies and drug stores in Cameroon and Nigeria. Health Policy Plan. 2015; 30: 1129–1141. doi: 10.1093/heapol/czu118 25339637 10.1093/heapol/czu118 PMC4597040

[8] Goodman C, Kachur SP, Abdulla S, Mwageni E, Nyoni J, Schellenberg JA, et al Retail supply of malaria-related drugs in rural Tanzania: risks and opportunities. Trop Med Int Health. 2004; 9: 655–663. doi: 10.1111/j.1365-3156.2004.01245.x 15189455 10.1111/j.1365-3156.2004.01245.x

[9] Whitty CJ, Chandler C, Ansah E, Leslie T, Staedke SG. Deployment of ACT antimalarials for treatment of malaria: challenges and opportunities. Malar J. 2008; 7 Suppl 1: S7. 19091041 10.1186/1475-2875-7-S1-S7 PMC2604871

[10] Kamal-Yanni MM, Potet J, Saunders PM. Scaling-up malaria treatment: a review of the performance of different providers. Malar J. 2012; 11: 414 doi: 10.1186/1475-2875-11-414 23231707 10.1186/1475-2875-11-414 PMC3547718

[11] Bruxvoort K, Kalolella A, Cairns M, Festo C, Kenani M, Lyaruu P, et al Are Tanzanian patients attending public facilities or private retailers more likely to adhere to artemisinin-based combination therapy? Malar J. 2015; 14: 87 doi: 10.1186/s12936-015-0602-x 25889767 10.1186/s12936-015-0602-x PMC4340668

[12] Briggs MA, Kalolella A, Bruxvoort K, Wiegand R, Lopez G, Festo C, et al Prevalence of malaria parasitemia and purchase of artemisinin-based combination therapies (ACTs) among drug shop clients in two regions in Tanzania with ACT subsidies. PLoS One. 2014; 9: e94074 doi: 10.1371/journal.pone.0094074 24732258 10.1371/journal.pone.0094074 PMC3986050

[13] Mangham LJ, Cundill B, Achonduh OA, Ambebila JN, Lele AK, Metoh TN, et al Malaria prevalence and treatment of febrile patients at health facilities and medicine retailers in Cameroon. Trop Med Int Health. 2012; 17: 330–342. doi: 10.1111/j.1365-3156.2011.02918.x 22098135 10.1111/j.1365-3156.2011.02918.x

[14] Arrow KJ, Panosian C, Gelband H editors. Saving lives, buying time: economics of malaria drugs in an age of resistance. Washington (DC), USA: National Academies Press; 2004. 25009879

[15] Sabot OJ, Mwita A, Cohen JM, Ipuge Y, Gordon M, Bishop D, et al Piloting the global subsidy: the impact of subsidized artemisinin-based combination therapies distributed through private drug shops in rural Tanzania. PLoS One. 2009; 4: e6857 doi: 10.1371/journal.pone.0006857 19724644 10.1371/journal.pone.0006857 PMC2730578

[16] Kangwana BP, Kedenge SV, Noor AM, Alegana VA, Nyandigisi AJ, Pandit J, et al The impact of retail-sector delivery of artemether-lumefantrine on malaria treatment of children under five in Kenya: a cluster randomized controlled trial. PLoS Med. 2011; 8: e1000437 doi: 10.1371/journal.pmed.1000437 21655317 10.1371/journal.pmed.1000437 PMC3104978

[17] Tougher S, ACTwatch Group, Ye Y, Amuasi JH, Kourgueni IA, Thomson R, et al Effect of the Affordable Medicines Facility--malaria (AMFm) on the availability, price, and market share of quality-assured artemisinin-based combination therapies in seven countries: a before-and-after analysis of outlet survey data. Lancet. 2012; 380: 1916–1926. doi: 10.1016/S0140-6736(12)61732-2 23122217 10.1016/S0140-6736(12)61732-2

[18] de Oliveira AM, Skarbinski J, Ouma PO. Performance of malaria rapid diagnostic tests as part of routine malaria case management in Kenya. Am J Trop Med Hyg. 2009; 80: 470–474. 19270300

[19] Baiden F, Webster J, Tivura M, Delimini R, Berko Y, Amenga-Etego S, et al Accuracy of Rapid Tests for Malaria and Treatment Outcomes for Malaria and Non-Malaria Cases among Under-Five Children in Rural Ghana. PloS One. 2012; 7: e34073 doi: 10.1371/journal.pone.0034073 22514617 10.1371/journal.pone.0034073 PMC3325982

[20] Cohen J, Fink G, Maloney K, Berg K, Jordan M, Svoronos T, et al Introducing rapid diagnostic tests for malaria to drug shops in Uganda: a cluster-randomized controlled trial. Bull World Health Organ. 2015; 93: 142–151.

[21] Uganda Bureau of Statistics and ICF International Inc. Uganda Demographic and Health Survey 2011. Kampala, Uganda: UBOS and Calverton, Maryland, USA: ICF International Inc; 2012.

[22] Hansen KS, Pedrazzoli D, Mbonye A, Clarke S, Cundill B, Magnussen P, et al Willingness-to-pay for a rapid malaria diagnostic test and artemisinin-based combination therapy from private drug shops in Mukono District, Uganda. Health Policy Plan. 2013; 28: 185–196. doi: 10.1093/heapol/czs048 22589226 10.1093/heapol/czs048 PMC3584993

[23] Mbonye AK, Magnussen P, Chandler CI, Hansen KS, Lal S, Cundill B, et al Introducing rapid diagnostic tests for malaria into drug shops in Uganda: design and implementation of a cluster randomized trial. Trials. 2014; 15: 303 doi: 10.1186/1745-6215-15-303 25069975 10.1186/1745-6215-15-303 PMC4125706

[24] Mbonye AK, Magnussen P, Lal S, Hansen KS, Cundill B, Chandler C, et al A cluster randomised trial introducing rapid diagnostic tests into the private health sector in Uganda: Impact on appropriate treatment of malaria. PLoS One. 2015; 10: e0129545 doi: 10.1371/journal.pone.0129545 26200467 10.1371/journal.pone.0129545 PMC4511673

[25] World Bank. World Bank Indicators. 2014; http://data.worldbank.org/indicator (accessed 30 October 2014).

[26] Walker D, Kumaranayake L. Allowing for differential timing in cost analyses: discounting and annualization. Health Policy Plan. 2002; 17: 112–118. 11861593 10.1093/heapol/17.1.112

[27] Management Sciences for Health. International Drug Price Indicator Guide 2011. Cambridge, Massachusetts, USA: Management Sciences for Health; 2012.

[28] Hayes RJ, Bennett S. Simple sample size calculation for cluster-randomized trials. Int J Epidemiol. 1999; 28: 319–26. 10342698 10.1093/ije/28.2.319

[29] Briggs A, Claxton C, Sculpher M. Decision modelling for health economic evaluation. Oxford, UK: Oxford University Press; 2006.

[30] Welton NJ, Sutton AJ, Cooper NJ, Abrams KR, Ades AE. Evidence synthesis for decision making in health care. Chichester, UK: John Wiley & Sons; 2012.

[31] Doubilet P, Begg CB, Weinstein MC, Braun P, McNeil BJ. Probabilistic sensitivity analysis using Monte Carlo simulation. A practical approach. Med Decis Making. 1984; 5: 157–177. 10.1177/0272989X8500500205 3831638

[32] Briggs A. Handling uncertainty in economic evaluation and presenting the results In: Drummond M, McGuire A, eds. Economic evaluation in health care. Oxford, UK: Oxford University Press; 2001.

[33] Fenwick E, O’Brien BJ, Briggs A. Cost-effectiveness acceptability curves--facts, fallacies and frequently asked questions. Health Econ. 2004; 13: 405–415. doi: 10.1002/hec.903 15127421 10.1002/hec.903

[34] Fenwick E, Claxton K, Sculpher M. Representing uncertainty: the role of cost-effectiveness acceptability curves. Health Econ. 2001; 10: 779–787. 11747057 10.1002/hec.635

[35] Batwala V, Magnussen P, Hansen KS, Nuwaha F. Cost-effectiveness of malaria microscopy and rapid diagnostic tests versus presumptive diagnosis: implications for malaria control in Uganda. Malar J. 2011; 10: 372 doi: 10.1186/1475-2875-10-372 22182735 10.1186/1475-2875-10-372 PMC3266346

[36] Ansah EK, Epokor M, Whitty CJ, Yeung S, Hansen KS. Cost-effectiveness analysis of introducing RDTs for malaria diagnosis as compared to microscopy and presumptive diagnosis in central and peripheral public health facilities in Ghana. Am J Trop Med Hyg. 2013; 89: 724–736. doi: 10.4269/ajtmh.13-0033 23980131 10.4269/ajtmh.13-0033 PMC3795104

[37] Mangham-Jefferies L, Wiseman V, Achonduh OA, Drake TL, Cundill B, Onwujekwe O, et al Economic evaluation of a cluster randomized trial of interventions to improve health workers’ practice in diagnosing and treating uncomplicated malaria in Cameroon. Value Health. 2014; 17: 783–791. doi: 10.1016/j.jval.2014.07.010 25498773 10.1016/j.jval.2014.07.010

[38] Matovu F, Nanyiti A, Rutebemberwa E. Household health care-seeking costs: experiences from a randomized, controlled trial of community-based malaria and pneumonia treatment among under-fives in eastern Uganda. Malar J. 2014; 13: 222 doi: 10.1186/1475-2875-13-222 24902959 10.1186/1475-2875-13-222 PMC4059171

[39] Hutchinson E, Chandler C, Clarke SE, Lal S, Magnussen P, Kayendeke M, et al “It puts life in us and we feel big”: Shifts in the local health care system during the introduction of rapid diagnostic tests for malaria into drug shops in Uganda. Crit Public Health. 2015; 25: 48–62. doi: 10.1080/09581596.2014.886762 25632175 10.1080/09581596.2014.886762 PMC4299853

