# Supplementary material for: Cost-effectiveness analysis of introducing malaria diagnostic testing in drug shops: A cluster-randomised trial in Uganda
Source: PLoS One. 2017 Dec 15;12(12):e0189758. doi: 10.1371/journal.pone.0189758 (PMC5731679; doi:10.1371/journal.pone.0189758)
Supplement: S1 Table — (DOCX) [file pone.0189758.s001.docx]

**S1 Table. Scenario analysis assuming identical malaria positivity rate and number of customers by study arm: costs and effects in a standard population of 1000 individuals suspected of malaria by study arm and incremental cost-effectiveness ratio (ICER) of replacing presumptive diagnosis by rapid diagnostic tests in drug shops in Mukono District, Uganda, 2011 (US$1=UGX2523).**

|  | --- mRDT arm --- | | -- Presumptive arm -- | |
| --- | --- | --- | --- | --- |
|  | ***N*** | ***%*** | ***N*** | ***%*** |
| *Individuals suspected of malaria* | *1000* | *100* | *1000* | *100* |
| True malaria ^#^ | 384 | 38 | 384 | 38 |
| Purchased ACT | 581 | 58 | 998 | 100 |
| Appropriately treated ^*^ | 737 | 74 | 385 | 38 |
|  | ***US$*** | ***%*** | ***US$*** | ***%*** |
| *Health sector cost per 1000 individuals* | *3217* | *34* | *2747* | *36* |
| Community sensitisation | 130 | 1 | 130 | 2 |
| Training of vendors | 642 | 7 | 534 | 7 |
| Supervision of vendors | 517 | 5 | 433 | 6 |
| mRDTs | 999 | 10 | 0 | 0 |
| ACTs | 929 | 10 | 1651 | 22 |
| *Household cost per 1000 individuals* | *6307* | *66* | *4868* | *64* |
| mRDTs (first visit) | 198 | 2 | 0 | 0 |
| ACTs (first visit) | 415 | 4 | 735 | 10 |
| Other drugs (first visit) | 2205 | 23 | 1101 | 14 |
| Fees, travel, food (first visit) | 581 | 6 | 562 | 7 |
| Drugs, fees, travel, food (subsequent visits) | 254 | 3 | 188 | 2 |
| Opportunity cost of time lost | 2655 | 28 | 2283 | 30 |
| *Total societal cost per 1000 individuals* | *9524* | *100* | *7615* | *100* |
| **Incremental analysis (Replace presumptive diagnosis by mRDT in 1000 individuals suspected of malaria)** |  |  |  |  |
| Incremental number of appropriately treated [95% CI] |  | 352 | [342; 362] |  |
| Incremental health sector cost, US$ [95% CI] |  | 470 | [454; 485] |  |
| Incremental societal cost, US$ [95% CI] |  | 1909 | [-9944; 13960] |  |
| ICER health sector perspective, US$ [95% CI] |  | 1.33 | [1.26; 1.41] |  |
| ICER societal perspective, US$ [95% CI] |  | 5.42 | [-28.23; 39.59] |  |

^#^ According to expert microscopy on a blood slide collected by the drug shop vendor at the time of consultation and blind reading later by the research team (reference diagnosis).

^*^ Individual with a positive reference diagnosis of malaria purchasing a course of ACT or an individual with a negative reference diagnosis not purchasing an ACT.
